# New Proteins from Cold: Antibiofilm Activity of Cold-Azurin and *Psy*Omp38 Against *Enterococcus faecalis* and *Klebsiella pneumoniae*

**DOI:** 10.1007/s12602-026-11009-7

**Published:** 2026-04-06

**Authors:** Martina Fionda, Marika Trecca, Diana Olimpo, Caterina D’Angelo, Maria Luisa Tutino, Orlando Donfrancesco, Michela Relucenti, Laura Selan, Marco Artini, Ermenegilda Parrilli, Rosanna Papa

**Affiliations:** 1 Department of Public Health and Infectious Diseases, Sapienza University, p.le Aldo Moro 5, Rome, 00185 Italy; 2 Department of Chemical Sciences, Federico II University, Complesso Universitario Monte S. Angelo, Via Cintia 4, Naples, 80126 Italy; 3 Department of Anatomy, Histology, Forensic Medicine and Orthopaedics, Sapienza University of Rome, Via Alfonso Borelli 50, Rome, 00161 Italy

**Keywords:** ESKAPE, Biofilm, Antivirulence, Antarctic proteins

## Abstract

In the present study, attention was given to *Enterococcus faecalis* and *Klebsiella pneumoniae* commonly causing healthcare-associated infections highly recalcitrant to antimicrobial therapy, also for their ability to form biofilms. These pathogens are often associated with a high risk of urinary tract infections due to biofilm-related complications in indwelling devices, commonly used in urological surgery. Currently, research is focusing on the development of new therapeutic strategies aimed at targeting bacterial virulence factors, such as biofilm, to reduce the increasing development of multidrug-resistant pathogens. In the present research, the activity of two antibiofilm proteins deriving from Antarctic marine bacteria, *Psy*Omp38 and Cold-Azurin, was evaluated on a collection of clinical *E. faecalis* and *K. pneumoniae* strains. In particular, the antibiofilm capabilities of *Psy*Omp38 and Cold-Azurin were tested against clinical bacterial strains characterized by different antimicrobial resistant profiles during distinctive phasis of biofilm development: initial attachment to the surface, pre-adhesion period and mature biofilm. This study provides interesting insights into the mechanism of action of the two proteins at different stages of the biofilm. Both proteins are able to impair, with different capabilities, biofilm formation of almost all bacterial tested strains. While Cold-Azurin disrupts mature biofilm only in a limited number of bacterial strains, *Psy*Omp38 shows disrupting activity against all *Klebsiella* strains and almost all *E. faecalis* strains. These findings suggest that Antarctic-derived proteins, particularly PsyOmp38, may represent promising candidates for the development of novel antibiofilm strategies against multidrug-resistant ESKAPE pathogens.

## Introduction

Over the years, an increasing number of pathogens have been reported as becoming multidrug-resistant (MDR) as a consequence of the misuse and overuse of antibiotics. A new report from the European Centre for Disease Prevention and Control (ECDC) ascribes over 35,000 deaths a year caused by antimicrobial resistant (AMR) microorganisms, accounting for EUR 1.4 billion in direct and indirect costs [1].

The development of AMR is a very complex mechanism, sometimes due to phenotypic variations of the microbial community rather than to the appearance of genetic mutations. A phenotypic variant related to AMR is certainly the ability of bacteria to form biofilms, in which they are incorporated into a matrix of self-produced extracellular polymeric substances. Many bacterial pathogens can form biofilms in vivo on biotic tissue and abiotic surfaces, such as implantable medical devices [2]. Biofilm formation is a physiological condition that provides bacteria with protection from the action of the immune system and antimicrobial agents. In addition, the horizontal transfer of genetic material between microbial cells within the biofilm is favoured, resulting in an increase in mutations that confer resistance to antibiotics. Hence, the biofilm can be defined as a virulence factor, since the infections that it sustains become chronic and are recalcitrant to conventional antibiotic therapies, often requiring invasive surgical intervention to clear the infection.

The US National Institutes of Health has reported that over 80% of microbial infections in the human body are associated with biofilms, many of which exhibit resistance to traditional antimicrobial treatments and often require surgical intervention to eradicate chronic infections. Consequently, biofilm-producing pathogens represent a significant threat to public health [3]. There is an urgent need to find new approaches for the prevention and treatment of microbial adhesion and biofilm formation [4]. Such approaches can target different stages of biofilm development [5].

Among biofilm-producing pathogens, the ESKAPE group represents one of the most clinically relevant threat. The term ESKAPE refers to Enterococcus faecium, Staphylococcus aureus, Klebsiella pneumoniae, Acinetobacter baumannii, Pseudomonas aeruginosa, and Enterobacter spp. These pathogens are responsible for severe and potentially life-threatening nosocomial infections, particularly among hospitalized patients [6, 7].

In this work, our attention focused in particular on two bacteria belonging to the ESKAPE group that are characteristic of urinary tract infections and are often found to be associated with each other: *E. faecalis* and *K. pneumoniae*. These two bacteria are responsible for healthcare-associated biofilm infections. Indeed, *K. pneumoniae* and *E. faecalis* are often associated with a high risk of urinary tract infections (UTIs) due to biofilm-related complications in indwelling devices, commonly used in urological surgery [8].

Several factors, including virulence determinants, biofilm formation, and antimicrobial resistance, contribute to the severity of infections caused by opportunistic pathogens such as *K. pneumoniae* [9] and *E. faecalis* [10]. Although both are human commensals - enterococci in the intestinal microbiota and *K. pneumoniae* on mucosal surfaces such as the gastrointestinal tract and oropharynx - they can cause severe and potentially life-threatening infections, particularly in hospitalised or immunocompromised patients.

Enterococci, particularly *E. faecalis* and *E. faecium*, are associated with infective endocarditis, urinary tract infections, catheter-related infections, and environmental faecal contamination [11]. Their ability to evade the host immune response and adhere to biotic and abiotic surfaces, mainly through biofilm formation, significantly enhances their persistence and antimicrobial resistance [12, 13]. Similarly, *K. pneumoniae* can systemically disseminate and cause pneumonia, urinary tract infections, bacteraemia, and liver abscesses. Its pathogenicity is largely mediated by key virulence factors, including the capsule, lipopolysaccharide (LPS), siderophores and fimbriae, the latter playing a crucial role in adhesion to surfaces and host tissues [14]. The growing antimicrobial resistance of both pathogens represents a major public health concern. In 2017, the World Health Organisation highlighted vancomycin-resistant *E. faecium* [13] and carbapenem-resistant *K. pneumoniae* [15] as priority pathogens, emphasising the urgent need to develop new antimicrobial strategies to combat these critical threats [16].

Currently, research is focusing on the development of new therapeutic strategies that target bacterial virulence factors to reduce the increasing development of multidrug-resistant pathogens. This approach could support a reduced use of broad-spectrum antibiotics and, consequently, limit the emergence of new resistant strains [17]. A promising strategy involves the identification of new anti-biofilm agents; in this context, microorganisms capable of thriving in extreme environments, such as Antarctica, are of particular interest [18]. Indeed, bacteria living in Polar regions established different survival strategies to live in extreme environmental conditions [18], these adaptations are often accompanied by modifications to both gene regulation and metabolic pathways, increasing the possibility of finding unique metabolites [19]. Furthermore, in environments where nutrients are extremely scarce or difficult to assimilate, Antarctic marine bacteria have developed survival strategies aimed at reducing the presence of competing microorganisms [20].

In recent papers, it has been demonstrated that Antarctic marine bacteria can produce anti-biofilm molecules active against the human pathogen *S. epidermidis* [21]. For example, the Antarctic marine bacterium *Pseudoalteromonas haloplanktis* TAC125 produces a long-chain fatty aldehyde, the pentadecanal endowed with strong anti-biofilm activity against this pathogen [22]. These anti-biofilm properties are not limited to *S. epidermidis*; Antarctic marine bacteria can reduce the biofilm formation of other pathogens, indeed, in a recent paper [23] the cell-free culture supernatants of four Antarctic marine bacteria resulted to be able to inhibit biofilm formation and disperse mature biofilm of ESKAPEs, suggesting potential activity against clinically relevant multidrug-resistant strains.

Building on these findings, this study focused on two Antarctic-derived proteins: Cold-Azurin [24] and *Psy*Omp38 [25], respectively produced by Antarctic marine bacteria *Pseudomonas* sp. TAE6080 [26] and *Psychrobacter* sp. TAE2020 [27]. Cold-Azurin showed antiadhesive and anti-biofilm activity against the opportunistic pathogen *S. epidermidis* [24, 26]. *Psy*Omp38, a component of the CATASAN complex composed of polysaccharides and the protein itself [27], has been recombinantly produced, purified, and characterized, showing the ability to inhibit adhesion, interfere with biofilm formation, and disrupt mature biofilms of *S. epidermidis* [25].

This study evaluated the antibiofilm activity of *Psy*Omp38 and Cold-Azurin against clinical *E. faecalis* and *K. pneumoniae* strains with different antimicrobial resistance profiles at various stages of biofilm development. Both proteins impaired biofilm formation in most strains, *Psy*Omp38 showed broader activity, including effective disruption of mature biofilms, whereas Cold-Azurin was effective mainly at earlier stages and against a limited number of strains.

These results expand current scientific knowledge on antibiofilm strategies and offer a solid basis for future developments aimed at improving the treatment of biofilm-associated infections, particularly those caused by multidrug-resistant pathogens.

## Materials and Methods

### Ethics Approval and Informed Consent

The following research was approved by the ethics committee of the Paediatric Hospital and Institute of Research Bambino Gesù (OPBG) in Rome, Italy (No. 1437_OPBG_2017 of July 2017). This study was conducted with respect to the Declaration of Helsinki as a statement of ethical principles for medical research involving human subjects. All participants and legal guardians signed an informed consent form that was included in the study.

### Bacterial Strains and Growth Conditions

In the present study, *E. faecalis* and *K. pneumoniae* bacterial species were used. For the species *E. faecalis* we used nine clinical strains and the reference strain *E. faecalis* ATCC 29,212, originally isolated from urine; for the species *K. pneumoniae* nine clinical strains and the *K. pneumoniae* ATCC 13,883 reference strain were analysed. Each strain was streaked onto a Petri dish containing Tryptone Soy Agar (TSA, Oxoid, Basingstoke, UK) and incubated under static conditions at 37 °C for 18 h. A single colony was inoculated into Brain Heart Infusion broth (BHI, Oxoid, Basingstoke, UK) and incubated overnight at 37 °C under constant agitation (180 rpm). The resistance profiles for each strain were performed according to the guidelines of Clinical Laboratory Standards Institute (CLSI, 2023) [23].

### Recombinant Production of Cold-Azurin

Antarctic azurin from *Pseudomonas* sp. TAE6080, was recombinantly produced as previously reported with slight modifications [24]. Briefly pET28b-Azu recombinant construct was transformed into the *E. coli* BL21(DE3) competent cells, plated on Luria-Bertani (LB) agar (Sigma Aldrich, Steinheim, Germany) with 50 µg/mL of kanamycin (Sigma Aldrich, Steinheim, Germany). After the overnight incubation of the plate, the transformed colonies were recovered and inoculated into 10 mL of LB broth without antibiotic at 30 °C, 180 rpm. Subsequently, 10 mL of inoculum was diluted in 100 mL of fresh LB medium containing 50 µg/mL of kanamycin and 5 µg/ml CuSO_4_ until reaching 0.5 OD at 30 °C, 180 rpm. The expression was induced by addition of 2 mM of isopropyl-1-thio-β-D-galactopyranoside (IPTG) to the culture. Bacterial cells were recovered after an overnight incubation and harvested by centrifugation at 4000 rpm at 4 °C. The bacterial pellet was washed with 150 mM of phosphate buffer pH 7, centrifuged at 4000 rpm for 10 min at 4 °C, and resuspended in an OSS solution (30 mM Tris-HCl pH 8, 20% sucrose,1 mM EDTA) necessary to form spheroplasts by osmotic shock. After 20 min of incubation at room temperature, the samples were centrifuged at 4000 rpm for 10 min at 4 °C, and the pellet was resuspended in 5 mM MgSO_4_. After incubation at 4 °C for 20 min and centrifugation at 10,000 rpm, the supernatant (periplasmic fractions) containing the recombinant azurin was collected and stored at − 20 °C until use (rPerAzu). The protein content was determined by Bradford assay.

### Purification of the Recombinant Cold-Azurin by Adsorption Chromatography

Cold-Azurin was purified from the periplasmic fraction (rPerAzu) by using adsorption chromatography. Amberlite XAD-2 polystyrene resin (Sigma Aldrich, Steinheim, Germany) was used for the purification [24]. 5.5 gr of the resin was activated with methanol, and after 1 h, it was packed into a glass column (11 cm by 1 cm). The stationary phase was washed with 3-bed volumes of water to remove the excess methanol and equilibrated with 3-bed volumes of 5 mM MgSO_4_. 7.55 mg of rPerAzu sample was added and incubated for 24 h under constant agitation at 60 rpm at 4 °C. The column was then washed with 3-bed volumes of 5 mM MgSO_4,_ and the elution of Cold-Azurin was subsequently carried out with methanol. The eluate obtained was collected, dried, and resuspended in a small volume (0.3 mL) of 0.02 M K_2_HPO_4_ pH 7 [24].

### Recombinant Production of PsyOmp38

The *Psychrobacter* sp. TAE2020 gene encoding Omp38 (WP_216581271) was amplified and cloned into the pET28a vector as previously described by Olimpo et al. (2025) [25]. The recombinant plasmid (pET28a-Omp38-SP) was transformed into *E. coli* BL21(DE3) competent cells, plated on LB agar supplemented with 50 µg/mL kanamycin (Sigma-Aldrich, Steinheim, Germany), and incubated overnight at 37 °C.

Single colonies were inoculated into 3 mL of LB medium containing 50 µg/mL kanamycin and incubated at 37 °C with shaking at 220 rpm for 24 h. The pre-culture was then diluted into 50 mL of fresh LB medium with 50 µg/mL kanamycin to an initial OD_600nm_= 0.1 and incubated under the same conditions. When the culture reached an OD_600nm_of 0.4, the temperature was reduced to 25 °C. Upon reaching an OD_600nm_of 0.6, protein expression was induced with 2 mM IPTG, and incubation was continued for 6 h at 25 °C.

### PsyOmp38 Recovery from Inclusion Bodies

Cells were collected and lysed by sonication using a Bandelin SONOPULS ultrasonic homogeniser equipped with an M72 probe (suitable for 2–30 mL volumes) at 35% amplitude for 30 min, applying 30 s on/off cycles. The lysates were centrifuged at 7000 × g for 30 min at 4 °C to separate the soluble (Sol) and insoluble (Insol) fractions [25].

Inclusion bodies were solubilised following McConnell and Pachón (2011) with slight modifications [28]. Briefly, the insoluble fractions were incubated in denaturation buffer at room temperature under gentle agitation. After incubation, the remaining insoluble material was removed by centrifugation at 6500 × g for 1 h at 4 °C.

For refolding experiments, the supernatant was dialysed against 50 mM Tris-HCl, pH 9.0, using 12 kDa molecular weight cut-off (MWCO) dialysis tubing (Sigma-Aldrich).

### PsyOmp38 Purification and Storage

The theoretical isoelectric point (pI) of *Psy*Omp38 was calculated as 4.46 using the ExPASy Compute pI/Mw tool. Therefore, refolding was performed in 50 mM Tris-HCl, pH 9.0, to ensure a net negative charge suitable for anion exchange chromatography.

Purification was carried out using Q Sepharose Fast Flow resin (Cytiva). Approximately 5 mg of renatured protein were loaded onto 4 mL of resin. The column was equilibrated sequentially with 10 column volumes (C.V.) of distilled water, elution buffer, and binding buffer. The renatured samples were applied for 2 h at room temperature under gentle agitation. Elution was performed using a NaCl gradient.

The eluted fractions were dialysed against distilled water, quantified, and lyophilised for storage.

### Biofilm Formation

#### Pre-Adhesion Period

Biofilm formation was analysed by the microtiter plate (MTP) biofilm assay [29]. The wells of a sterile 96-well polystyrene flat base plate were filled with 100 µl of BHI medium in the presence and absence of the samples to be examined. Specifically, the substances examined separately are Cold-Azurin at a concentration of 0.25 µg/µL [24], and the *Psy*Omp38 protein at a concentration of 0.01 µg/µL [25]. The bacteria were added to the wells at a 1:100 dilution of the overnight inoculum. The experiment was conducted in three replicates for each strain. The plates were incubated overnight under static conditions at 37 °C. The following day, the supernatant containing planktonic cells was gently removed, the plates were washed with double-distilled water and carefully dried by placing them in an inverted position. 100 µl of 0.1% (w/v) crystal violet was added to the wells. The plates were incubated for 15 min at room temperature, then washed with water and dried. The adherent biofilm was solubilised with 20% (v/v) glacial acetic acid and 80% (v/v) ethanol, and after 10 min of incubation, the total biofilm biomass was quantified using a spectrophotometer (Tecan Männedorf, Switzerland) at 590 nm [30].

#### Mature Biofilm

The activity of Cold-Azurin and *Psy*Omp38 on the preformed biofilm was also analysed. The experiment was conducted in three replicates for each strain using a sterile 96-well flat-bottomed polystyrene plate. The wells were filled with 100 µl of BHI containing a 1:100 dilution of the overnight inoculum, and the plates were incubated at 37 °C for 24 h under static conditions. The following day, the content of the wells was aspirated, and a BHI medium solution containing and not containing the protein was added. Specifically, Cold-Azurin was used at a concentration of 0.25 µg/µL [24], while the *Psy*Omp38 protein was tested at a concentration of 0.1 µg/µL [25]. The plates were incubated for another 24 h at 37 °C under static conditions. The following day, the plates were analysed as described previously [30].

### SEM Analysis

Bacterial biofilm samples grown on aluminium stubs were processed as follows. First, they were fixed in 2.5% glutaraldehyde in 0.1 M phosphate buffer (PB), pH 7.4, for at least 48 h. The samples were then washed overnight in PB. The following day, the post fixation procedure was carried out using 1.33% osmium tetroxide (OsO₄) for 1 h at room temperature. After a 20-min rinse with distilled water, the samples were treated with 1% tannic acid in water for 30 min. Another 20-min wash with distilled water followed. Excess moisture was gently removed using filter paper. The samples were then mounted on specimen holders using a carbon film and examined using a Hitachi SU3500 scanning electron microscope (Hitachi, Japan) under variable pressure conditions (7 kV, 30 Pa) [31, 32]. Digital images were artificially coloured to highlight specific features and facilitate interpretation.

### Surface Coating Assay

The antiadhesive activity was assessed as previously reported [25, 28]. After depositing and drying the protein sample at a concentration of 0.1 µg/µL for *Psy*Omp38 and 0.78 µg/µL for Cold-Azurin on the surface, bacterial culture was added, and biofilm formation was assessed by crystal violet staining. The presence of a clear area at the deposition site indicates inhibition of adhesion.

### Eukaryotic Cells

The adenocarcinomic human alveolar basal epithelial cells A549 (ATCC CRM-CCL-185) were cultured in minimal essential medium with Earle’s balanced salt solution high glucose 4.5 g/l (MEM/EBSS), supplemented with 10% foetal calf serum (FCS), 1% glutamine and 1% penicillin–streptomycin in an atmosphere of 5% CO_2_ at 37 °C. All media were from Euroclone (Milan, Italy). Confluent monolayers were used 24 h after seeding.

### Cell Viability

The cytotoxicity of Cold-Azurin on adenocarcinomic human alveolar basal epithelial cells (A549) was assessed using an MTT cell proliferation kit (Roche Applied Science, Penzberg, Germany). A549 cells were incubated at 37 °C under 5% CO_2_ for 2, 4 and 24 h after cell inoculation as above described. A 50 µl volume of MTT working solution was added to each well and the mixture was incubated for additional 4 h. Purple crystal formazan was observed around cells at 40X magnification under a microscope. The cell medium was carefully removed, and then 100 µl of dimethyl sulfoxide (DMSO) was added to each well to dissolve formazan. After 15 min of incubation at 37 °C to completely dissolve formazan, the absorbance at 490 nm was measured on Tecan Infinite 200pro microplate. Cell survival was expressed as a percentage of viable cells in the presence of Cold-Azurin at a concentration of 0.25 µg/µL, in comparison to control cells. Student’s T-test was performed for statistical analysis.

### Statistical Analysis of Biological Evaluation

Data reported were statistically validated using Student’s *t*-test comparing the mean absorbance of treated and untreated samples. The significance of differences between mean absorbance values was calculated using a two-tailed Student’s *t*-test. A *p-value* < 0.05 was considered significant.

## Results

### Effect of Cold-Azurin on Biofilm Produced by *E. faecalis*

Previous studies found that Cold-Azurin [24] and *Psy*Omp38 [25] proteins showed excellent antibiofilm activity against the opportunistic pathogen *Staphylococcus epidermidis*. Therefore, in the present study, the antibiofilm activity of these proteins was investigated against two bacterial pathogen species belonging to the ESKAPE group: Gram-negative *K. pneumoniae* and Gram-positive *E. faecalis*.

Antimicrobial susceptibility of clinical and reference strains of *E. faecalis* species is reported in Table 1.

**Table 1:** Antimicrobial susceptibility of clinical and reference strains of *E. faecalis* species [23]

	Carbapenems	Fluoroquinolones		Glycopeptides		Tetracyclines	Oxazolidinones	Resistance Phenotype
	IM	LEV	CIP	TEC	VAN	TGC	LNZ	
	(10 µg)	(5 µg)	(5 µg)	(30 µg)	(5 µg)	(15 µg)	(10 µg)	
*E. faecalis* ATCC29121	I	S	S	S	S	S	R	WT
EF1	I	S	S	S	S	S	R	WT
EF2	I	S	S	S	S	S	S	WT
EF3	I	S	S	S	S	S	R	WT
AC1	I	S	S	S	S	S	R	WT
189	I	R	R	S	S	S	R	WT
190	I	R	R	S	S	S	S	WT
222	I	S	S	S	S	S	R	WT
239	I	S	S	S	S	S	R	WT
6015	I	R	R	S	S	S	R	WT

Antimicrobial susceptibility was performed according to the guidelines of EUCAST Clinical Breakpoint Tables v. 13.0 (valid from 1 January 2023). IM: imipenem; LEV: levofloxacin; CIP: ciprofloxacin; TEC: teicoplanin; VAN: vancomycin; TGC: tigecycline; LNZ: linezolid. S: sensitive; R: resistant; I: susceptible with increased exposure. WT: absence of phenotypically detectable resistance to all antimicrobial categories.

Firstly, the biofilm-forming ability of each bacterial strain was determined at 24 h and 48 h. Results are reported in Table 2.

**Table 2:** Biofilm production of *E. faecalis* clinical strains and *E. faecalis* ATCC 29,212

Bacterial strain	Biofilm 24 h (OD 590 nm)	Biofilm 48 h (OD 590 nm)
*E. faecalis* ATCC 29,212	0.734 ± 0.019	0.732 ± 0.063
EF1	0.584 ± 0.028	0.994 ± 0.028
EF2	0.526 ± 0.021	0.519 ± 0.011
EF3	0.440 ± 0.013	0.636 ± 0.076
AC1	0.935 ± 0.091	1.495 ± 0.066
189	1.463 ± 0.014	1.848 ± 0.086
190	1.283 ± 0.031	1.803 ± 0.212
222	0.637 ± 0.018	0.779 ± 0.026
239	0.678 ± 0.040	1.383 ± 0.068
6015	1.512 ± 0.026	1.728 ± 0.173

All *E. faecalis* strains resulted to be strong biofilm producers.

For these experiments, Cold-Azurin was recombinantly expressed in *E. coli* and purified as previously reported [24]. To exclude any possible effect of the proteins on bacterial viability, Cold-Azurin and *Psy*Omp38 were analysed also for antimicrobial activity. Results did not highlight any antimicrobial activity on tested strains.

The anti-biofilm effects of Cold-Azurin and *Psy*Omp38 were evaluated on ESKAPE pathogens either during biofilm development by adding them to the medium at the beginning of the growth (time zero, pre-adhesion period), or after biofilm formation (after 24 h of bacterial culture, mature biofilm). The biofilm in untreated samples was grown in BHI broth supplemented with the corresponding buffer used for each protein solubilization (0.02 M K_2_HPO_4_ pH 7 for Cold-Azurin and 50 mM Tris-HCl at pH 9 for *Psy*Omp38, respectively), as control. In such cases the buffer had a slight effect in the total amount of biofilm production.

**Fig. 1:**
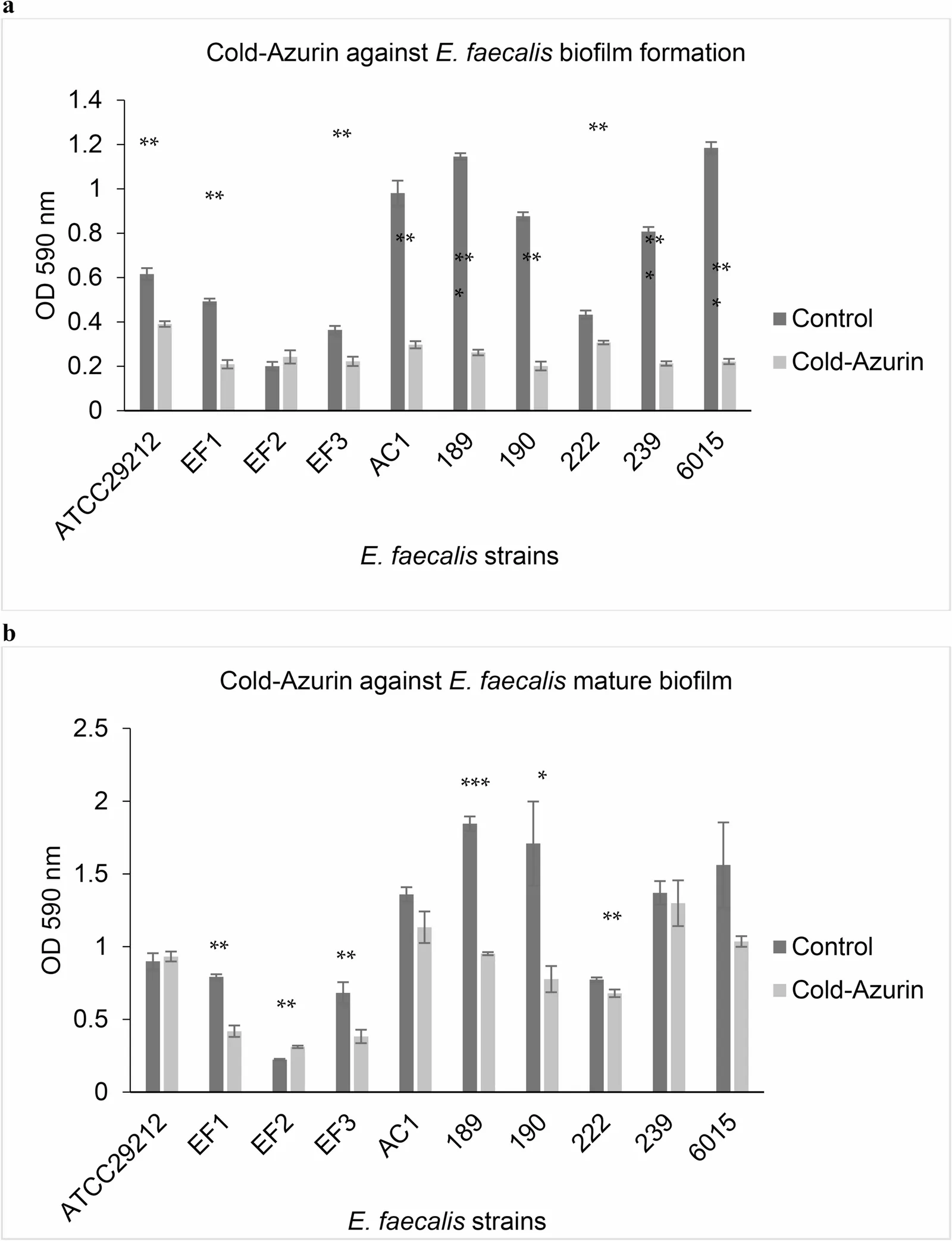
Effect of cold-azurin against biofilm formation of different clinical *E. faecalis* strains and *E. faecalis* ATCC 29212 reference strain. **a** Effect on biofilm formation. Cold-azurin was added at a concentration of 0.25 µg/µL to the culture medium at time zero (0 h, pre-adhesion period) and the biofilm was analysed after overnight incubation. The ordinate axis represents the biofilm biomass expressed as optical density at 590 nm. **b** Effect of cold-azurin on the mature biofilm. Cold-azurin was added at a concentration of 0.25 µg/µL to the culture medium after 24 h of biofilm growth (24 h of bacterial culture) and the biofilm was analysed after overnight incubation. The ordinate axis represents the biofilm biomass expressed as optical density at 590 nm.Data are expressed as biofilm biomass quantified by optical density at 590 nm after 24h of treatment with cold-azurin. The control experiment was performed in BHI broth supplemented with the 0.02 M K2HPO4 pH 7, used for cold-azurin resuspension. Each data point is composed of three independent experiments, each performed with at least three replicates. Error bars indicate the standard deviations of all the measurements. Statistical analysis was determined with Student’s t-test (*p <0.05; **p < 0.01; ***p <0.001)

The effect of Cold-Azurin on biofilm formation was strongly strain-dependent (Fig. 1a). However, Cold-Azurin exerted a strong inhibitory activity against *E. faecalis* biofilm formation on nine out of ten tested strains. In six strains, the percentage of biofilm reduction was higher than 50% compared to the untreated sample. In ATCC 29,212 and clinical strain 222 the residual biofilm production ranged between 20% and 40%. Only in the case of clinical strain EF-2, the presence of Cold-Azurin induced a slight and not statistically significant increase in biofilm production.

The activity of Cold-Azurin was also analysed on mature biofilm. As shown in Fig. 1b, Cold-Azurin disassembled the preformed biofilm in six out of nine clinical tested strains. For clinical EF-1, EF-3, 189, and 190 strains, the ability of Cold-Azurin to disaggregate mature biofilm was higher than 40%. An exception was observed with clinical EF2 strain, where the treatment led to an increase of about 40% of biofilm. For *E. faecalis* ATCC 29,212, the addition of protein did not affect mature biofilm. It is worth noting that the biofilm developed after 48 h of growth is more abundant and more difficult to eradicate.

Data are expressed as biofilm biomass quantified by optical density at 590 nm after 24 h of treatment with Cold-Azurin. The control experiment was performed in BHI broth supplemented with the 0.02 M K_2_HPO_4_ pH 7, used for Cold-Azurin resuspension. Each data point is composed of three independent experiments, each performed with at least three replicates. Error bars indicate the standard deviations of all the measurements. Statistical analysis was determined with Student’s *t*-test (*p < 0.05; **p < 0.01; ***p < 0.001).

### Effect of PsyOmp38 on Biofilm Produced by *E. faecalis*

For these experiments, *Psy*Omp38 were recombinantly expressed in *E. coli* and purified as previously reported [25]. We analysed the effect of *Psy*Omp38 on *E. faecalis* biofilm formation. *Psy*Omp38 was tested at a concentration of 0.01 µg/µL for pre-adhesion assay and a ten-fold higher concentration (0.1 µg/µL) for the mature biofilm disaggregation assay, since mature biofilms are more difficult to eradicate.

**Fig. 2:**
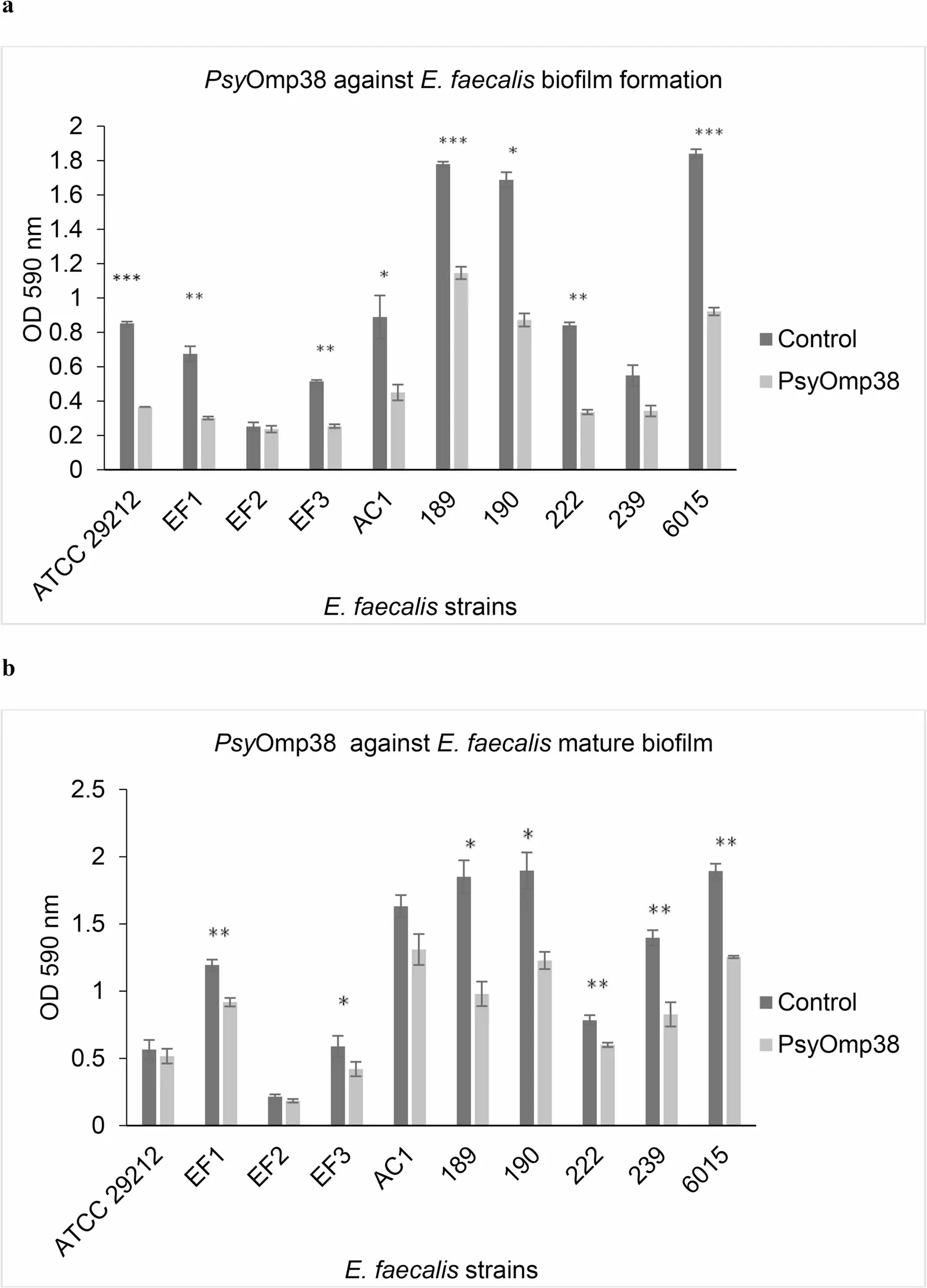
Effect of PsyOmp38 against biofilm formation of different clinical *E. faecalis* strains and *E. faecalis* ATCC 29212 reference strain. **a** Effect on biofilm formation. PsyOmp38 was added at a concentration of 10 µg/µL to the culture medium at time zero (0 h, pre-adhesion period), and the biofilm was analysed after overnight incubation. The ordinate axis represents the biofilm biomass expressed as optical density at 590 nm. **b** Effect of PsyOmp38 on the mature biofilm. PsyOmp38 was added at a concentration of 100 µg/µL to the culture medium after 24 h of biofilm growth (24 h of bacterial culture), and the biofilm was analysed after overnight incubation. The ordinate axis represents the biofilm biomass expressed as optical density at 590 nm. Data are expressed as biofilm biomass quantified by optical density at 590 nm after 24h of treatment with PsyOmp38. The control experiment was performed in BHI broth supplemented with 50 mM Tris-HCl at pH 9, used for PsyOmp38 resuspension. Each data point is composed of three independent experiments, each performed with at least three replicates. Error bars indicate the standard deviations of all the measurements. Statistical analysis was determined with student’s t-test (*p <0.05; **p < 0.01; ***p <0.001)

Figure 2a shows that the *Psy*Omp38 protein reduced biofilm formation in nine out of ten clinical *E. faecalis* strains examined. Specifically, the inhibition of biofilm formation was in the range of approximately 40% to 60%.

A promising result was also obtained by analysing the activity of *Psy*Omp38 on mature biofilm. For all *E. faecalis* strains, a good biofilm-disrupting activity of *Psy*Omp38 was observed, especially for the clinical strain 189, which exhibited a 50% reduction compared to the untreated control (Fig. 2b).

The ordinate axis represents the biofilm biomass expressed as optical density at 590 nm.

Data are expressed as biofilm biomass quantified by optical density at 590 nm after 24 h of treatment with *Psy*Omp38. The control experiment was performed in BHI broth supplemented with 50 mM Tris-HCl at pH 9, used for *Psy*Omp38 resuspension. Each data point is composed of three independent experiments, each performed with at least three replicates. Error bars indicate the standard deviations of all the measurements. Statistical analysis was determined with Student’s *t*-test (*p < 0.05; **p < 0.01; ***p < 0.001).

### Ultrastructural Morphology of *E. faecalis* Following the Treatment with Cold-Azurin and PsyOmp38

Ultrastructural morphology of *E. faecalis* biofilm was observed using a variable pressure scanning electron microscope (VP-SEM). This analysis was performed on the AC1 clinical strain treated or not-treated with Cold-Azurin and *Psy*Omp38, being this strain a strong biofilm producer responsive to the action of both proteins and it showed a good initial attachment to the substrate (Fig. 7).

**Fig. 3:**
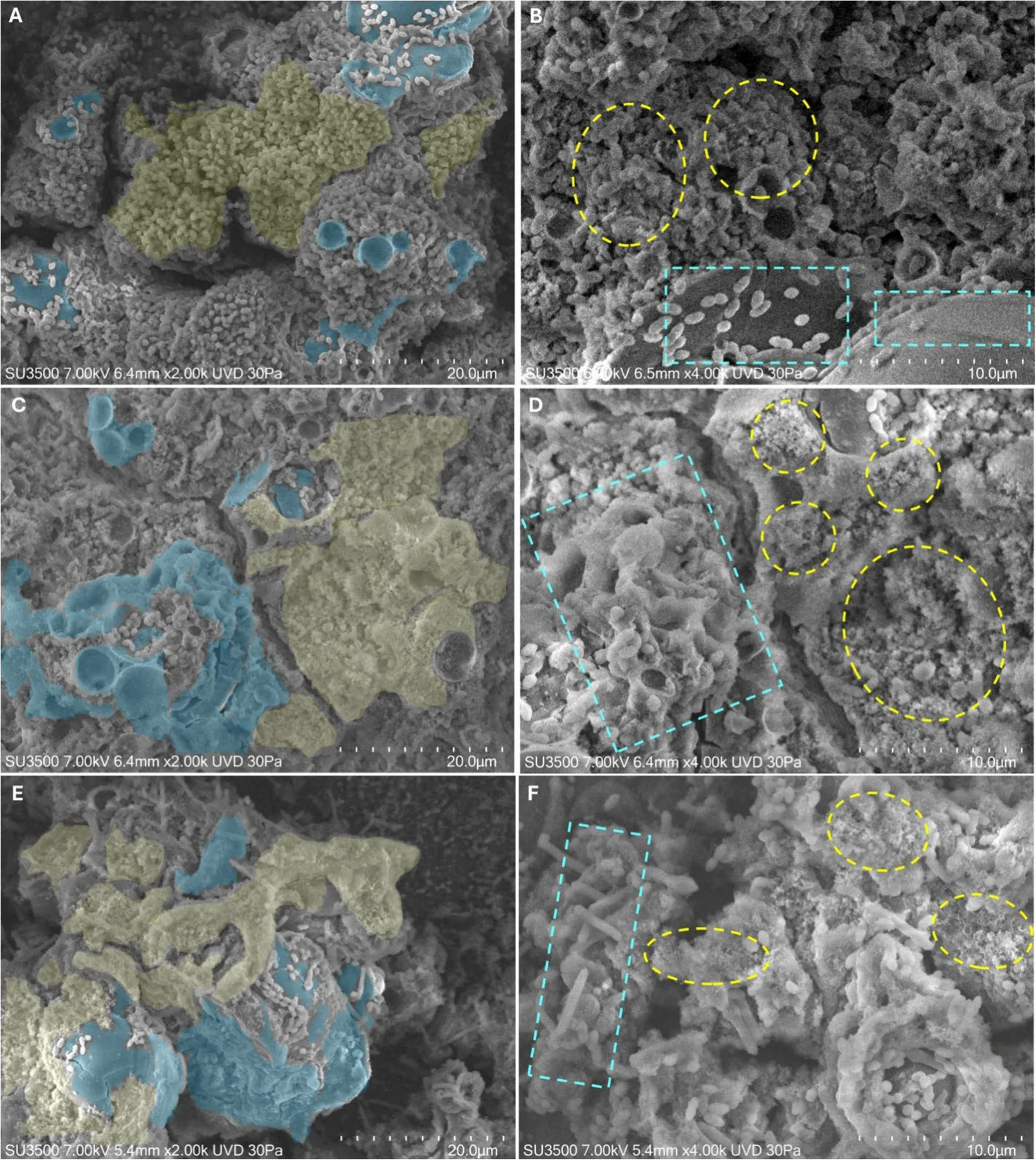
VP-SEM images of *E. faecalis*. **a**, **b** Untreated *E. faecalis* AC1. Smooth and dense areas in the biofilm are artificially coloured in light blue, and spongy areas are coloured in light yellow. Magnification 2000X bar 20 micron **a**. Blue rectangles emphasise biofilm dense and smooth areas, yellow circles emphasise spongy areas, magnification 4000X bar 10 micron **b**. **c**, **d** *E. faecalis* AC1 treated with cold-azurin. Artificially coloured in light blue are the compact, dense and smooth areas in the biofilm, whereas the spongy areas are represented in light yellow. Magnification 2000X bar 20 micron **c**. magnification 4000X bar 10 micron **d**. **e**, **f** *E. faecalis* AC1 treated with PsyOmp38. Light blue represents the dense and compact areas of the biofilm, while light yellow emphasises the spongy areas. Magnification 2000X bar 20 micron **e**. Magnification 4000X bar 10 micron **f**

Under VP-SEM, the biofilm of *E. faecalis* appears as a dense matrix composed of extracellular polymeric substances (EPS) interspersed with bacterial cells (Fig. 3a). Within this matrix, regions of variable density are observed: some areas appear compact and tightly packed (blue), while others exhibit a more porous, sponge-like architecture (yellow) (Fig. 3b). At higher magnification (Fig. 3b), the spongy regions reveal a micrometric trabecular arrangement of the EPS, forming a three-dimensional canalicular network. Upon treatment with Cold-Azurin (Figs. 3c, d), morphological alterations become evident. The EPS trabeculae in the spongy zones show signs of flaking, and at increased magnification, these trabeculae are seen to consist of a fibrous scaffold of thin filaments forming a sub-micrometric meshwork (Figs. 3c, d). At higher magnification is evident that the treatment with Cold-Azurin affects only the spongy areas of the biofilm (yellow circles); the compact areas are not affected (blue rectangles). EPS trabeculae appear emptied of their amorphous component, while their fibrillar component is visible, arranged in a three-dimensional network with submicrometric meshes. The amorphous component of the EPS appears partially removed by the action of Cold-Azurin.

In the sample treated with *Psy*Omp38 (Figs. 3e, f), the structural integrity of the EPS trabeculae is further disrupted. Figure 3f highlights pronounced flaking, with the fibrous network becoming increasingly disaggregated and the filaments more diffusely distributed. At higher magnification, it is possible to appreciate that the treatment with *Psy*Omp38 affects only the spongy area trabeculae. They appear to have a structure made up of a 3D meshwork of very fine fibrillary components, and the amorphous matrix appears to have been removed by *Psy*Omp38 treatment.

### Effect of Cold-Azurin on Biofilm Produced by *K. pneumoniae*

Antimicrobial susceptibility of clinical and reference strains of *K. pneumoniae* is reported in Table 3.

**Table 3:** Antimicrobial susceptibility of clinical and reference strains of *K. pneumoniae* species [23]

	Penicillins		Cephalosporins		Carbapenems	Monobactams	Fluoroquinolones	Aminoglycosides	Miscellaneous	Resistance Phenotype
	AMP	AMC	FOX	CRO	IM	ATM	CIP	AK	SXT	
	(10 µg)	(30 µg)	(30 µg)	(30 µg)	(10 µg)	(30 µg)	(5 µg)	(30 µg)	(25 µg)	
*K. pneumoniae* **ATCC13883**	R	S	S	S	S	S	R	S	S	WT
**1-KPC**	R	R	R	R	S	R	R	S	R	MDR
**2-KPC**	R	R	R	R	R	R	R	R	S	MDR
**3-KPC**	R	R	R	R	R	R	R	R	S	MDR
**4-KPC**	R	R	R	R	R	R	R	R	S	MDR
**5-KPC**	R	R	R	R	R	R	R	R	R	PDR
**KPC31-1**	R	R	S	R	S	R	R	S	R	MDR
**KPC31-2**	R	R	S	R	S	R	R	S	R	MDR
**KPC-3R**	R	R	R	R	R	R	R	R	R	PDR
**KPC-C4**	R	S	S	R	S	R	R	S	R	MDR

Antimicrobial susceptibility was performed according to the guidelines of EUCAST Clinical Breakpoint Tables v. 13.0 (valid from 1 January 2023). AMP: ampicillin; AMC: amoxicillin and clavulanic acid; FOX: cefoxitin; CRO: ceftriaxone; IM: imipenem; ATM: aztreonam; CIP: ciprofloxacin; AK: amikacin; SXT: trimethoprim/sulfamethoxazole. S: sensitive; R: resistant; I: susceptible with increased exposure.

WT: absence of phenotypically detectable resistance to all antimicrobial categories; MDR: acquired non-susceptibility to at least one agent in three or more antimicrobial categories; PDR: non-susceptibility to all agents in all antimicrobial categories.

Regarding *Klebsiella*, except for the *K. pneumoniae* ATCC13883 strain, all the other strains were strong biofilm producers (Table 4).

**Table 4:** Biofilm production of *K. pneumoniae* clinical strains and ATCC 13,883

Bacterial strain	Biofilm 24 h (OD 590 nm)	Biofilm 48 h (OD 590 nm)
*K. pneumoniae* ATCC 13,883	0.143 ± 0.012	0.179 ± 0.040
1KPC	1.056 ± 0.036	1.142 ± 0.072
2KPC	0.614 ± 0.065	0.459 ± 0.046
3KPC	1.230 ± 0.126	1.650 ± 0.117
4KPC	0.698 ± 0.035	0.475 ± 0.046
5KPC	0.796 ± 0.071	0.606 ± 0.058
KPC 31 − 1	0.691 ± 0.021	0.556 ± 0.047
KPC 31 − 2	0.652 ± 0.045	0.574 ± 0.028
KPC 3R	0.191 ± 0.009	0.256 ± 0.051
KPC C4	1.541 ± 0.072	1.826 ± 0.079

The biofilm in untreated samples was grown in BHI broth supplemented with the corresponding buffer used for each protein solubilization (0.02 M K_2_HPO_4_ pH 7 for Cold-Azurin and 50 mM Tris-HCl at pH 9 for *Psy*Omp38, respectively), as control.

**Fig. 4:**
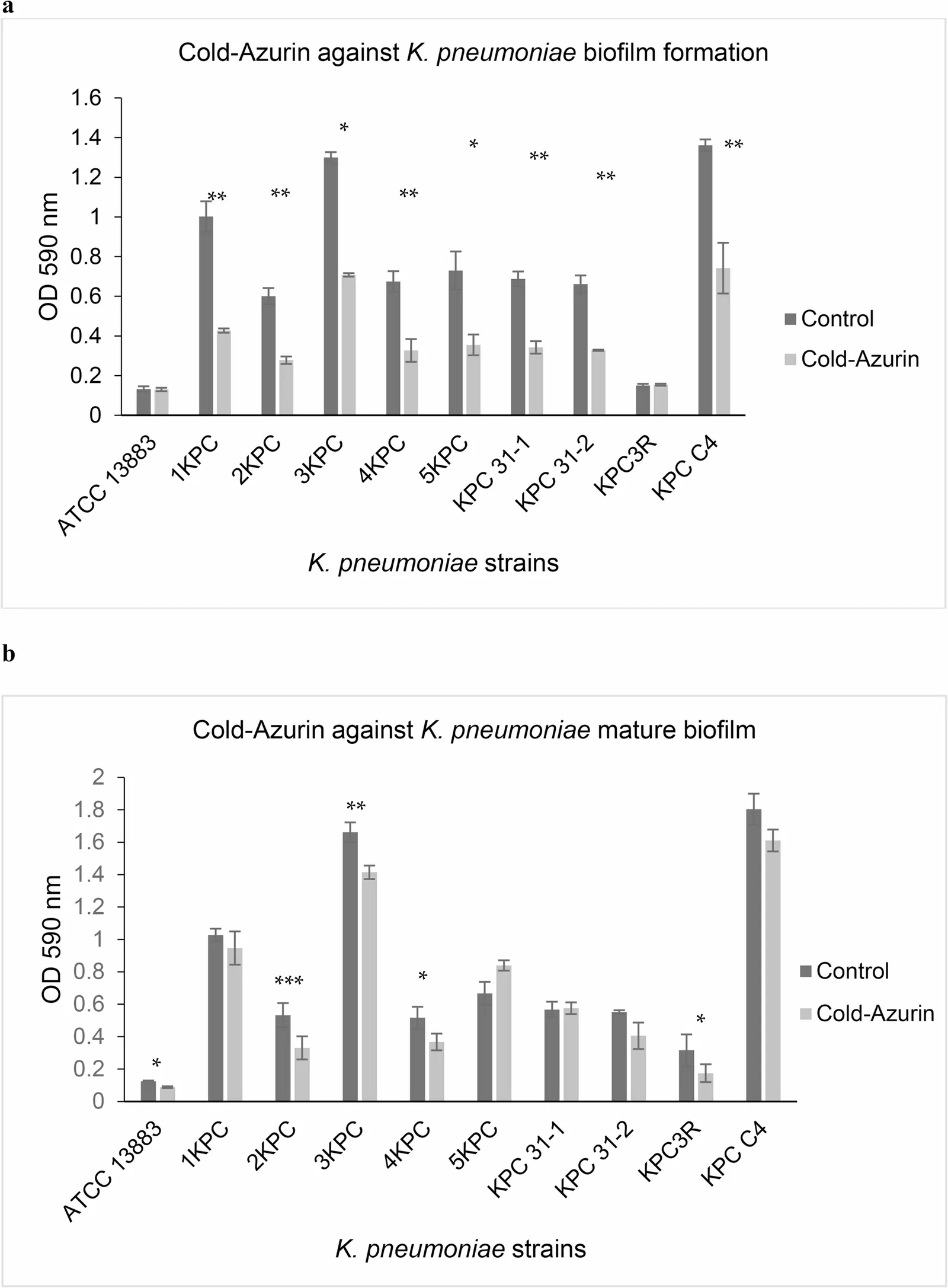
Effect of cold-azurin against biofilm formation of different clinical strains and *K. pneumoniae* ATCC 18883 reference strain of *K. pneumoniae* species. **a** Effect on biofilm formation. Cold-azurin was added at a concentration of 0.25 µg/µL to the culture medium at time zero (0 h, pre-adhesion period), and the biofilm was analysed after overnight incubation. The ordinate axis represents the biofilm biomass expressed as optical density at 590 nm. **b** Effect of cold-azurin on the mature biofilm. Cold-azurin was added at a concentration of 0.25 µg/µL to the culture medium after 24 h of biofilm growth (24 h of bacterial culture), and the biofilm was analysed after overnight incubation. The ordinate axis represents the biofilm biomass expressed as optical density at 590 nm. Data are expressed as biofilm biomass quantified by optical density at 590 nm after 24h of treatment with cold-azurin. The control experiment was performed in BHI broth supplemented with the 0.02 M K2HPO4 pH 7, used for Cold-azurin resuspension. Each data point is composed of three independent experiments, each performed with at least three replicates. Error bars indicate the standard deviations of all the measurements. Statistical analysis was determined with Student’s t-test (*p < 0.05; **p < 0.01; ***p < 0.001)

Cold-Azurin inhibited biofilm formation by approximately 40% to 60% in eight out of nine clinical strains tested (Fig. 4a). For *K. pneumoniae* ATCC 13,883 and KPC-3R strains no variation in biofilm formation in the presence of Cold-Azurin compared to the untreated control was observed.

Figure 4b shows the activity of Cold-Azurin on mature *K. pneumoniae* biofilm. A slight biofilm disaggregation was observed in almost all clinical strains analysed after 24 h of incubation with the protein. The best result was observed for the clinical strain KPC-3R which showed approximately 40% biofilm disaggregation, contrasting with the biofilm formation data on this strain, where Cold-Azurin had no effect.

Data are expressed as biofilm biomass quantified by optical density at 590 nm after 24 h of treatment with Cold-Azurin. The control experiment was performed in BHI broth supplemented with the 0.02 M K_2_HPO_4_ pH 7, used for Cold-Azurin resuspension. Each data point is composed of three independent experiments, each performed with at least three replicates. Error bars indicate the standard deviations of all the measurements. Statistical analysis was determined with Student’s *t*-test (*p < 0.05; **p < 0.01; ***p < 0.001).

### Effect of PsyOmp38 on Biofilm Produced by *K. pneumoniae*

Clinical and reference *K. pneumoniae* strains were also tested for biofilm formation in the presence of *Psy*Omp38 protein.

**Fig. 5:**
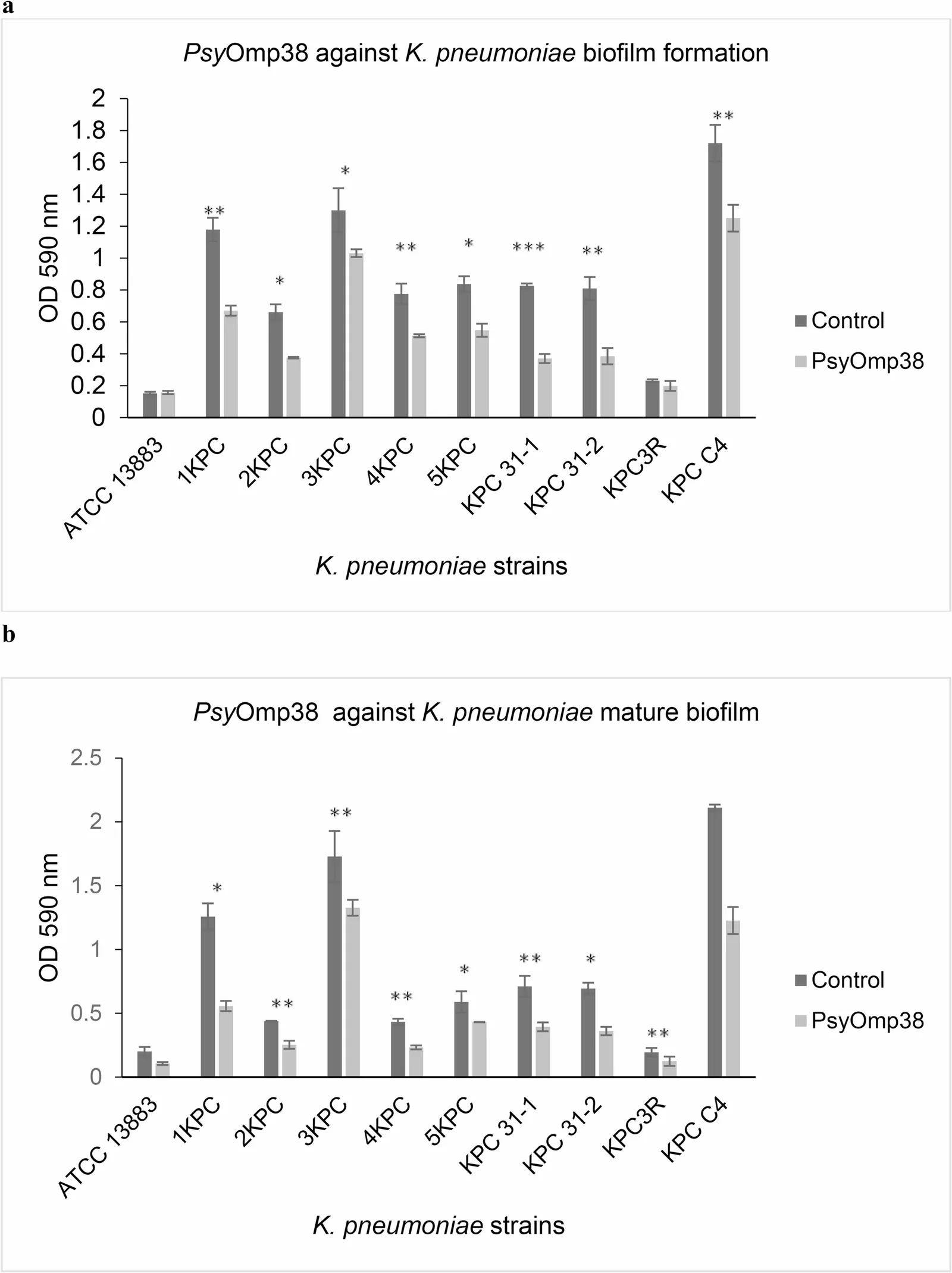
Effect of PsyOmp38 against biofilm formation of different clinical strains and *K. pneumoniae* ATCC 18883 reference strain of *K. pneumoniae*. **a** Effect on biofilm formation. PsyOmp38 was added at a concentration of 10 µg/µL to the culture medium at time zero (0 h, pre-adhesion period), and the biofilm was analysed after overnight incubation. The ordinate axis represents the biofilm biomass expressed as optical density at 590 nm. **b** Effect of PsyOmp38 on the mature biofilm. PsyOmp38 was added at a concentration of 100 µg/µL to the culture medium after 24 h of biofilm growth (24 h of bacterial culture), and the biofilm was analysed after overnight incubation. The ordinate axis represents the biofilm biomass expressed as optical density at 590 nm. Data are expressed as biofilm biomass quantified by optical density at 590 nm after 24h of treatment with PsyOmp38. The control experiment was performed in BHI broth supplemented with 50 mM Tris-HCl at pH 9, used for PsyOmp38 resuspension. Each data point is composed of three independent experiments, each performed with at least three replicates. Error bars indicate the standard deviations of all the measurements. Statistical analysis was determined with Student’s t-test (*p < 0.05; **p < 0.01; ***p < 0.001)

Figure 5a shows a good antibiofilm activity on all the analysed clinical strains. Best results were observed for KPC 31 − 1 and KPC31-2 clinical strains, with a significant reduction in biofilm formation of approximately 60% compared to the untreated control. No effect on the *K. pneumoniae* ATCC 13,883 reference strain was observed.

Biofilm-disrupting activity of *Psy*Omp38 was observed for all *K. pneumoniae* clinical strains (Fig. 5b). This is a particularly relevant result, considering the high resistance of the mature biofilm. As shown in Fig. 5b, biofilm disruption ranged from approximately 20% to 60%.

### Ultrastructural Morphology of *K. pneumoniae* Following the Treatment with Cold-Azurin and PsyOmp38

**Fig. 6:**
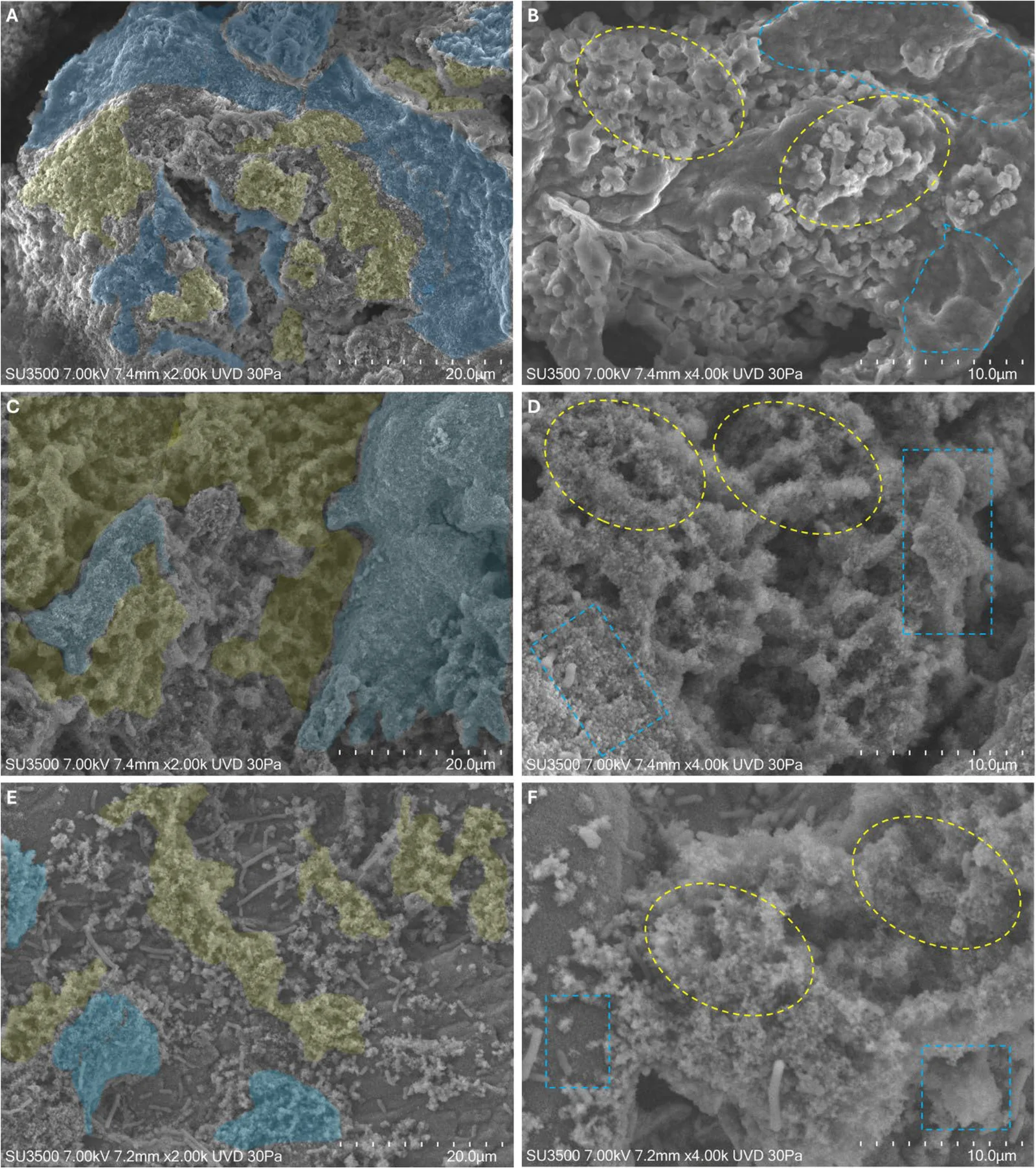
VP-SEM images of *K. pneumoniae*. **a**, **b***K. pneumoniae* 1KPC untreated sample. Biofilm spongy areas are coloured in light yellow. Compact and dense areas are artificially coloured in light blue. Magnification 2000X bar 20 micron **a**. The blue rectangles emphasise the dense and smooth areas in the biofilm, yellow circles instead emphasise the spongy areas, magnification 4000X bar 10 micron **b**. **c**, **d***K. pneumoniae* 1KPC treated with cold-azurin. Artificially coloured in light blue are the compact, dense and smooth areas in the biofilm, whereas the spongy areas are represented in light yellow. Magnification 2000X bar 20 micron **c**. Images at higher magnification show that the treatment with cold-azurin affects only the trabeculae in the spongy areas of the biofilm (yellow circles); whereas the compact areas are not affected (blue rectangles). EPS trabeculae appear emptied of their amorphous component, while their fine fibrillar component is well visible, arranged in a three-dimensional network with submicrometric meshes. magnification 4000X bar 10 micron **d**. **e**, **f*** K. pneumoniae* 1KPC treated with PsyOmp38. Light blue depicts the dense and compact areas of the biofilm, while the light-yellow colour emphasises the spongy areas. Magnification 2000X bar 20 micron **e**. Pictures at higher magnification allow to appreciate that the treatment with PsyOmp38 affects only the spongy area trabeculae. This treatment has a stronger effect with respect to the cold-azurin treatment, the trabeculae show a finest fibrillary structure concerning the cold-azurin treatment in **d**, and the amorphous matrix appears in large part dissolved by the PsyOmp38 treatment. Magnification 4000X bar 10 micron **f**

Ultrastructural morphology of *K. pneumoniae* biofilm was performed on 1KPC clinical strain treated or not-treated with Cold-Azurin and *Psy*Omp38. VP-SEM reveals that the *K. pneumoniae* biofilm forms a dense aggregate of extracellular polymeric substances (EPS), within which individual bacterial cells are embedded. Similar to *E. faecalis*, *Klebsiella* biofilm exhibits regions of varying density, with compact zones (blue) interspersed with more porous, sponge-like areas (yellow). At higher magnification (Fig. 6b), the EPS in these spongy regions (yellow circles) is organised into a micrometric trabecular network, within which a three-dimensional system of canaliculi extends-often containing bacterial cells. Treatment with Cold-Azurin (Figs. 6c, d) induces morphological alterations, particularly within the spongy regions of the EPS. The trabeculae appear to flake, and at greater magnification, they are revealed to consist of a fibrous scaffold of thin filaments forming a sub-micrometric mesh, from which the amorphous component has been partially removed by Cold-Azurin. The disintegration of the EPS structure is even more pronounced in samples treated with *Psy*Omp38 (Figs. 6e, f). The trabeculae exhibit more extensive flaking, and the filamentous scaffold appears further disrupted and finely dispersed, indicating a more significant loss of the amorphous matrix.

### Surface Coating Assay

**Fig. 7:**
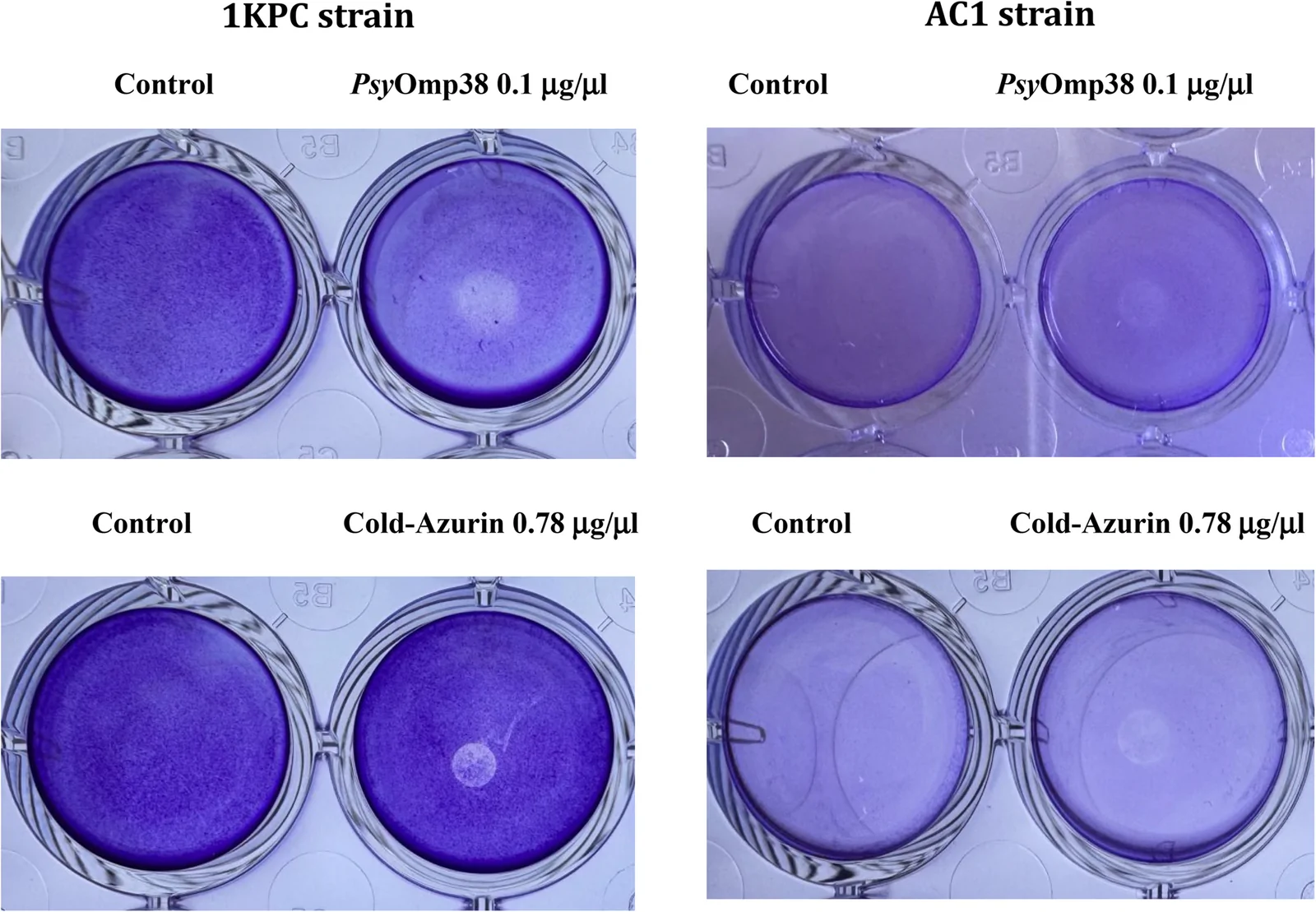
The effect of cold-azurin and PsyOmp38 functionalization on the initial attachment of *K. pneumoniae* 1KPC1 and *E. faecalis* AC1

Given the antibiofilm properties shown by Cold-Azurin and *Psy*Omp38 at the beginning of the growth (time zero, pre-adhesion period), both proteins were tested for their ability to impair the initial attachment of *E. faecalis* and *K. pneumoniae* strains to the polystyrene surface, by coating assay (Fig. 7). The surface coating assay was performed by using AC1 and 1KPC1 strains, which had previously been ultrastructurally characterized by VP-SEM.

Both proteins altered the surface properties, reducing the initial attachment of tested bacterial strains. The central area of the bottom of a well was coated by the deposition of a single drop (2 µl) of each protein, and the ability of the coated surfaces to repel biofilm formation by *E. faecalis* AC1 and *K. pneumoniae* 1KPC strains was tested. As shown in Fig. 7, biofilm formation was repelled exclusively in the protein-coated regions.

Analysis of the surfactant capability was evaluated after incubation for 1 h on polystyrene plates previously functionalized with Cold-Azurin and*Psy*Omp38 solutions. The centre of each well of a 24-well tissue-culture-treated polystyrene microtiter plate was coated with 2 µl of 0.78 mg/ml of Cold-Azurin and 0.1 mg/ml of *Psy*Omp38 solutions, respectively. Biofilm formation was evaluated after 24 h incubation using crystal violet staining and then photographed. The areas where biofilm was not present appear unstained.

### Effect of Cold-Azurin and PsyOmp38 on Eukaryotic Cell Viability

**Fig. 8:**
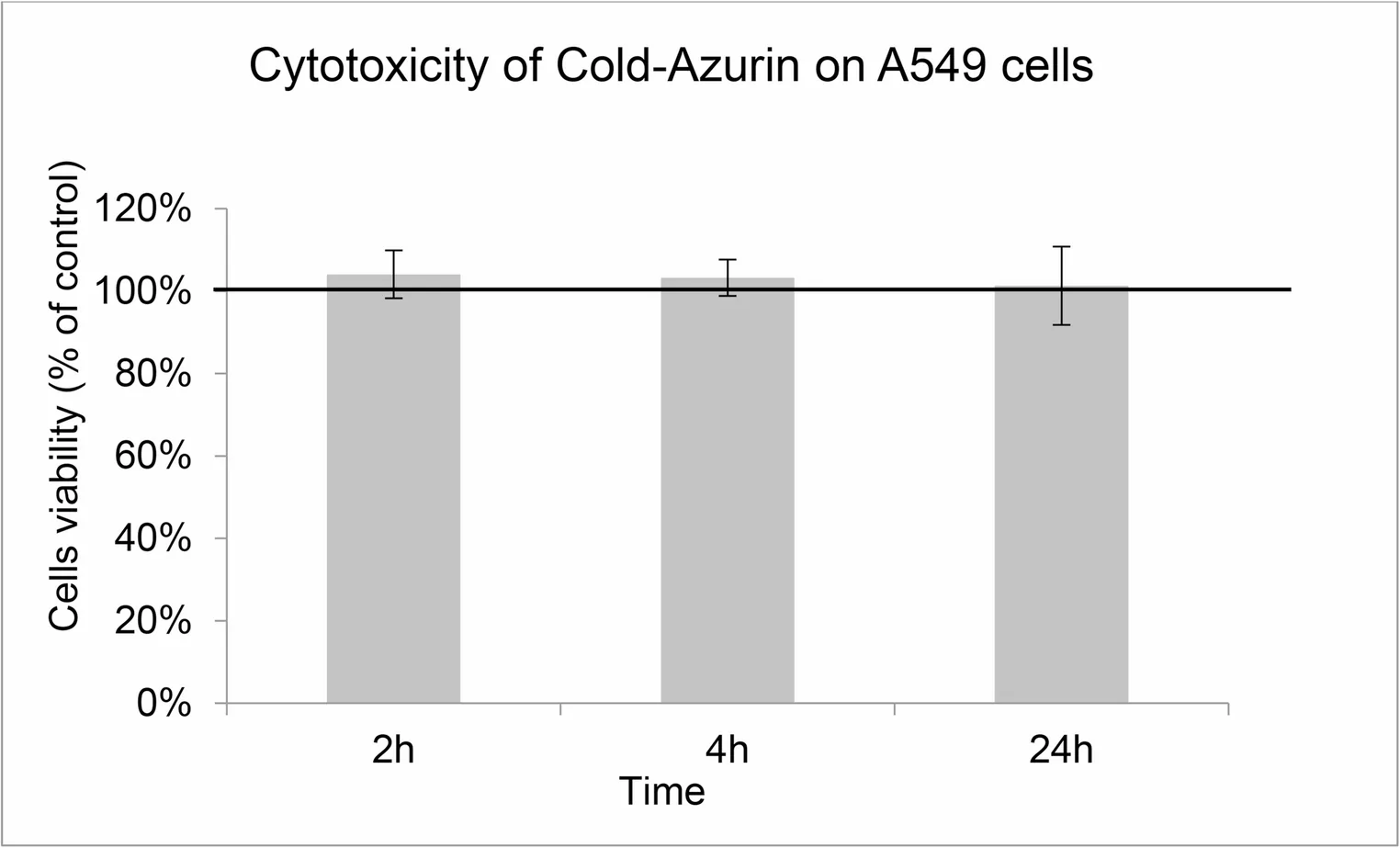
Effect of cold-azurin on A549 immortalized cell line. Cells were incubated for 2, 4 and 24 h with 0.25 µg/µL of protein. Cell viability was assessed by the MTT assay. Cell survival was expressed as a percentage of viable cells in the presence of cold-azurin in comparison to control cells, as described in the Materials and Methods section. Values are given as means ± SD (*n* = 6) with respect to control cells

To further investigate a possible medical application of Cold-Azurin and *Psy*Omp38 their biocompatibility was assessed. *Psy*Omp38 cytocompatibility was already evaluated on two immortalised eukaryotic cell lines showing no cytotoxic effect at concentration used in this work [25]. The biocompatibility of Cold-Azurin was here investigated performing a cytotoxic assay on adenocarcinomic human alveolar basal epithelial cells A549. The cell viability was observed after 2, 4 and 24 h of incubation with protein at a concentration of 0.25 µg/µL used for microbiological assays. As shown in Fig. 8, the presence of Cold-Azurin did not impair cell viability and cell morphology of A549 showed no evident changes (data not shown).

Effect of Cold-Azurin on A549 immortalized cell line. Cells were incubated for 2, 4 and 24 h with 0.25 µg/µL of protein. Cell viability was assessed by the MTT assay. Cell survival was expressed as a percentage of viable cells in the presence of Cold-Azurin in comparison to control cells, as described in the Materials and methods section. Values are given as means ± SD (*n* = 6) with respect to control cells.

## Discussion

This work focuses on evaluating the antibiofilm properties of two proteins against two different bacterial targets. This approach might seem somewhat broad and not entirely linear. Why not concentrate on a single protein or a single pathogen? The rationale is twofold. First, we aimed to demonstrate that the two proteins, previously shown to be active against *S. epidermidis*, are also active against two ESKAPE strains, a Gram-negative and a Gram-positive bacterium. Second, we sought to gain insight into their mechanisms of action by comparing the results across these two distinct strains. The first objective is crucial for envisioning the future use of these two proteins not only against single-species infections but also in complex multi-species infections. The second objective contribute to better understanding of the mechanisms underlying their antibiofilm activity, to make them more effective and targeted.

On *S. epidermidis*, both Cold-Azurin and *Psy*Omp38 proved to be very effective in preventing biofilm formation and similarly resulted in being active on the target pathogens used in this study. Surprisingly, the two antibiofilm proteins were effective despite the profound differences in the biofilm composition formed by these three microorganisms. The extracellular matrix of *S. epidermidis* biofilms is composed of polysaccharide intercellular adhesin (PNAG/PIA) [33], extracellular DNA [34], and proteinaceous factors such as Aap and Embp [35], which together mediate cell–cell adhesion and structural stability. In *K. pneumoniae*, biofilm matrices are rich in strain-specific exopolysaccharides composed of galactose, glucose, mannose, or uronic acid residues [36]. This bacterium does not have a single dominant polysaccharide like PNAG in *S. epidermidis*, but rather several strain-specific matrix polysaccharides [37]. In the case of *Enterococcus faecalis*, biofilms incorporate extracellular DNA, exopolysaccharides and surface proteins [38].

The results that both antibiofilm proteins were efficient regardless of the differences in the biofilm composition could suggest that the same antibiofilm protein can act through different mechanisms and/or that the target of its action is not a single component of the EPS but rather involves multiple components. Therefore, it becomes even more difficult to infer a single underlying mechanism responsible for the antibiofilm activity. Unless this is a generic effect associated with the physicochemical properties of the two proteins, which in some way hinder biofilm formation by interfering with its structuring, despite the different composition of the matrix. SEM analyses also seem to indicate that the presence of the two different antibiofilm proteins during the formation phase leads to the development of compromised biofilms, exhibiting comparable structural alterations with respect to the untreated samples. These observations suggest that Cold-Azurin and PsyOmp38 induce similar phenotypic outcomes at the biofilm level under the tested conditions.

suggesting that the mechanism underlying the antibiofilm action could actually be the same. One characteristic shared by the two proteins, that may contribute to interference with biofilm structuring is their amphipathic nature. Indeed, both Cold-Azurin [39] and *Psy*Omp38 [25] contain hydrophilic regions and hydrophobic patterns within their three-dimensional structures. This feature could interfere with the organisation of the biofilm matrix, making the interactions among the various matrix components less stable. Moreover, the presence of hydrophobic regions common to both proteins could explain their ability to interact with the polystyrene surface, thereby reducing bacterial attachment during the early stages of biofilm formation. This broad-spectrum activity on the initial adhesion and biofilm formation stages suggests the possibility of using the antibiofilm proteins for the prevention of multispecies infections associated with medical devices. In the context of the present work, the report data support the use of materials derivatised with the Cold-Azurin or *Psy*Omp38 to prevent catheter-urinary tract infections. Notably, for *Psy*Omp38, the possibility of producing silicone materials coated with this protein by simple casting has already been demonstrated [25].

The situation is completely different in the case of mature biofilm disruption. The mechanisms underlying this activity are different from those involved in interference with the biofilm formation phase, and the data presented in this work are consistent with this behaviour. Indeed, in some cases, antibiofilm proteins are active against biofilm formation but ineffective against mature biofilms, and vice versa. Furthermore, the results obtained do not suggest a generic mechanism that is effective against all analysed strains. Cold-Azurin, as shown in previous studies [24], is not able to disrupt *S. epidermidis* mature biofilms and, as demonstrated in this work, it is effective only against some *Enterococcal* strains and a few *Klebsiella* strains. In contrast, *Psy*Omp38 shows activity against mature biofilms formed by all *Klebsiella* tested strains and is ineffective only against a single Enterococcal strain.

## Conclusions

The current investigation identifies two important antibiofilm proteins produced by Antarctic bacteria, effective against two pathogenic bacteria, such as *K. pneumoniae* and *E. faecalis*, both associated with a rapid development of AMR in hospital settings. Beyond the well-characterised genetic determinants contributing to antibiotic resistance in these bacterial species, the biofilm phenotype represents a crucial factor in the establishment and persistence of antimicrobial resistance. This study provides interesting insights into the mechanism of action of the two proteins at different stages of the biofilm. Both proteins impaired biofilm formation of almost all bacterial tested strains and showed high biocompatibility on eukaryotic cell lines. While Cold-Azurin disrupts mature biofilm only in a limited number of bacterial strains, *Psy*Omp38 shows disrupting activity against all *Klebsiella* strains and almost all *Enterococcal* strains.

For *Psy*Omp38 is therefore possible to propose a combined use with traditional antibiotics to treat biofilm-related infections, to combine antibiofilm activity with antimicrobial activity, thereby making the antibiotic effective also against already established biofilm. However, the present study is limited to in vitro analyses, which cannot fully reproduce the complexity of the biofilm-associated infection in vivo. In addition, the molecular mechanisms underlying the antibiofilm activity of these proteins were not fully elucidated. Future research should therefore focus on investigating the mechanism of action of these proteins, as well as evaluating their efficacy in more complex experimental models. Furthermore, in vivo investigations and clinical studies will be necessary to fully assess the therapeutic potential, optimal delivery strategies, and long-term efficacy of these antibiofilm proteins in the treatment of biofilm-associated infections. Overall, Antarctic-derived antibiofilm proteins may represent a promising new avenue for the development of strategies aimed to controlling persistent biofilm-associated infections.

## Data Availability

No datasets were generated or analysed during the current study.
